# Evolving Effect of the COVID-19 Pandemic on Cancer-Related Encounters

**DOI:** 10.1200/CCI.21.00200

**Published:** 2022-03-08

**Authors:** Jack W. London, Elnara Fazio-Eynullayeva, Matvey B. Palchuk, Christopher McNair

**Affiliations:** ^1^ Department of Cancer Biology, Sidney Kimmel Cancer Center, Thomas Jefferson University, Philadelphia, PA; ^2^ TriNetX LLC, Cambridge, MA

## Abstract

This is an update to a previously published report characterizing the impact that efforts to control the COVID-19 pandemic have had on the normal course of cancer-related encounters.

**METHODS:**

Data were analyzed from 22 US health care organizations (members of the TriNetX global network) having relevant, up-to-date encounter data. Although the original study compared encounter data pre–COVID-19 (January-April 2019) with the corresponding months in 2020, this update considers data through April 2021. As before, cohorts were generated for all neoplasm patients (malignant, benign, in situ, and of unspecified behavior), all new incidence neoplasm patients, exclusively malignant neoplasm patients, and new incidence malignant neoplasm patients. Data on the initial cancer stage were available for calendar year 2020 from about one third of the study's organizations.

**RESULTS:**

Although COVID-19 cases fluctuated through 2021, newly diagnosed cancers closely paralleled the prepandemic base year 2019. Similarly, screening for breast, colorectal, and cervical cancers quickly recovered beginning in May 2020 to prepandemic numbers. Preliminary data for the initial cancer stage showed no significant difference (*P* > .10) in distribution for breast or colon cancers between 2019 and 2020.

**CONCLUSION:**

Although the number of COVID-19 cases fluctuated, the steep declines observed during March and April 2020 in screening for breast and colon cancer and patients with newly diagnosed cancer did not continue through the rest of 2020 and into April 2021. Screening and new incidence cancer numbers quickly rose compared with prepandemic levels. The concern that more patients with advanced-stage cancer would be seen in the months following the drastic dips of March-April 2020 was not realized as the major disruption to normal cancer care was limited to these 2 months.

## INTRODUCTION

The deadly COVID-19 pandemic has disrupted lives worldwide. Cases were first seen in large numbers in the United States in March and April 2020. Our group performed and published a study^1^ to quantify the immediate impact that COVID-19 had on deviation from normal cancer care activities, including cancer screening efforts. We used encounter data from 20 health care institutions to compare numbers of patient encounters in early 2019 with those occurring between January and April 2020 as COVID-19 became a pandemic. This study considered a variety of patient cohorts including those with existing cancer, those new incidences of disease, those with specific types of cancer, and those simply undergoing screenings. A significant decline was found in the number of patient encounters in this early COVID-19 period across the groups studied, notably for those with new incidence of cancer. Of the cancer types analyzed, lung, colorectal, and hematologic cancer cohorts exhibited decreases in patient encounters, roughly averaging 39%, whereas decreases for breast cancer, prostate cancer, and melanoma ranged from almost 48% to nearly 52%. In addition, cancer screenings declined drastically, with both breast cancer and colorectal cancer screenings dropping by well more than 80%.

**CONTEXT**

**Key Objective**
This study quantifies the evolving effects that the COVID-19 pandemic has had on cancer screening and initial diagnosis following our previous study (after April 2020) by using data from a network of 22 US institutions consisting of more than 34 million patients.
**Knowledge Generated**
Using prepandemic 2019 as a basis for comparison, we show that significant declines in cancer screening and new patient diagnosis were primarily seen in the first 2 months of the pandemic. From May 2020 through April 2021, screening and new incidence cancer numbers quickly rose compared with prepandemic levels.
**Relevance**
The early concern that more advanced-stage cancers would be initially seen as the pandemic continued was not realized. However, because of the recent emergence of COVID-19 variants that stress medical resources and affect public behavior, there is a continued need to monitor pandemic effects on patients with cancer.

Subsequently, other studies reported quantified negative effects on cancer care and diagnosis on the basis of other data sources. Patt et al^2^ notably used medical claims data to show the delay in cancer diagnosis and treatment for American seniors. Concern was raised at that time that a continuation of these interruptions to the normal course of cancer diagnosis would result in later-stage initial presentations and subsequent poorer prognoses for some patients. This apprehension over future cancer occurrences included a warning from the Director of the National Cancer Institute.^3^

This communication updates our previously reported results with metrics on patients having cancer-related encounters in the months of May 2020 through April 2021 of the COVID-19 pandemic in the United States. We were able to leverage an existing health research network platform to analyze data from 22 different provider institutions across the United States representing more than 34 million patients and having immediate data on relevant patient encounters. (This represents an additional two institutions that were not in our original study.) We focused on patients seen at these health care organizations (HCOs) with diagnostic codes for malignant, benign, in situ, and unspecified neoplasms (ICD-10 C00-D49). We looked at the number of these patients having encounters in May 2020 through April 2021, compared with the same months in our prepandemic base year, 2019. We further focused on the subset of these patients having malignant neoplasm diagnoses (ICD-10 C00-C96 and D37-D49). We also obtained the number of these patients (all neoplasms and malignant-only) being seen for the first time at these institutions—possibly for screening, initial diagnosis, second opinion, or treatment initiation.

Furthermore, we investigated the initial diagnostic stage of newly diagnosed patients for calendar year 2020 compared with the base year 2019. These stage data are typically acquired from tumor registry data rather than hospital electronic medical record (EMR) system sources. There is typically a lag of months in the reporting of registry data, so these stage data were only available for 2020 and only from seven of the 22 organizations. We compared the initial stage of patients with breast and colon cancer in 2020 with patients with the same diagnoses in 2019 to determine if more advanced-stage patients were seen.

## METHODS

### COVID and Cancer Research Network Creation

Data for this study were obtained from a subset of HCOs that are members of the TriNetX Research Network. TriNetX^4^ is a global federated health research network providing access to aggregate data from EMRs including demographics, diagnoses, procedures (including cancer screening examinations), medications, laboratory testing, vital signs, and genomic information. Additional data on patients with cancer from tumor registries and results of genomic testing are integrated with the EMR data on the TriNetX platform. The network consists of academic medical centers, community hospitals, and physician practices.

The COVID and Cancer Research Network (CCRN) subnet of our initial study was formed of 20 HCOs (28 million patients) having immediate, relevant data, ie, reliable, comprehensive data on the diagnoses of patients with encounters at the organization for the time period January 1, 2019, through April 30, 2021. This primarily means that the HCO had established data acquisition for the TriNetX platform as of January 1, 2019, and had refreshed these data so that encounters through April 30, 2021, would be included. The current study uses an *updated COVID and Cancer Research Network* (uCCRN), composed of 22 institutions representing data from more than 34 million patients. Because of the lag of tumor registry data entry, staging data (which typically reside in these registries rather than hospital EMRs) were only reliably available through December 2020 and only from seven HCOs.

### Cohort Definitions

Patients studied were those patients with HCO encounters who had diagnostic ICD-10 codes for neoplasms (ICD-10 C00-D49). This includes patients with benign, in situ, and unspecified neoplasms, along with malignant neoplasms. A subgroup of patients was defined as those who had no previous encounter with the HCO related to the indicated diagnosis (defined as new incidence encounters). These new incidence patients might have been seen at the HCO for screening, initial diagnosis, second opinion, or treatment initiation.

### Cohort Comparison and Analysis

For all groups, patient cohort sizes were obtained by constructing TriNetX platform queries with the appropriate diagnosis (ICD-10), procedure (ICD-10, CPT), and date constraints. Although our previous study considered data by month (ie, January, February, March, and April 2020), this update looks at the data at 2-week intervals (eg, May 1-May 15, May 16-May 30, and May 31-June 14, 2020). The platform then returns patient cohort data for these queries. Patients satisfying a query in multiple instances (eg, multiple encounters in the time period) are counted only once. Percent change between each month was calculated by subtracting the patient count in 2019 from the same time interval in 2020 and 2021 and dividing by the total patient count from 2019.

## RESULTS

### Updated Network Characteristics

**FIG 1.**
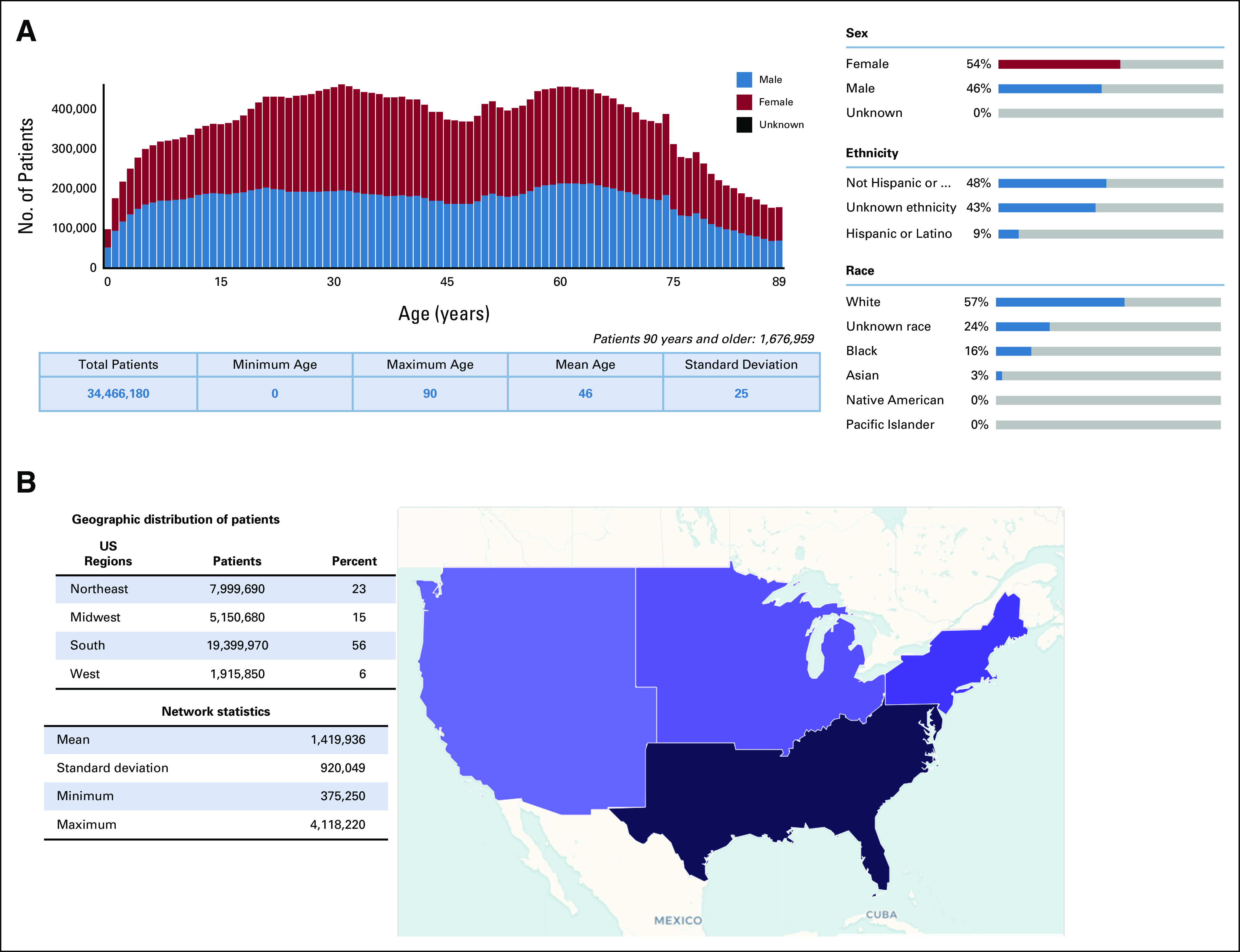
The COVID-19 cancer research network descriptive statistics and demographics: (A) distribution of patient age, sex, and race/ethnicity within the network and (B) geographic distribution split into Northeast, Midwest, South, and West regions. Network statistics represent the distribution in sizes of individual institutions included in the network.

Patients in the uCCRN were 54% female and 46% male, with a mean age of 46 years. Although 9% of the uCCRN patients were identified as Hispanic or Latino, 43% of the network patients had an unknown ethnicity. Similarly, although 19% of the network were non-White races, 24% lacked a racial identity, as some institutions lack complete demographic information on their patient population (Fig 1A). Geographically, patients were primarily from health care institutions located in the Southern United States, representing 56% (more than 19 million patients) of the network with the Northeast contributing 23%, the Midwest 15%, and the West 6% (Fig 1B).

### New Incidence Patient Trends (all neoplasms)

**FIG 2.**
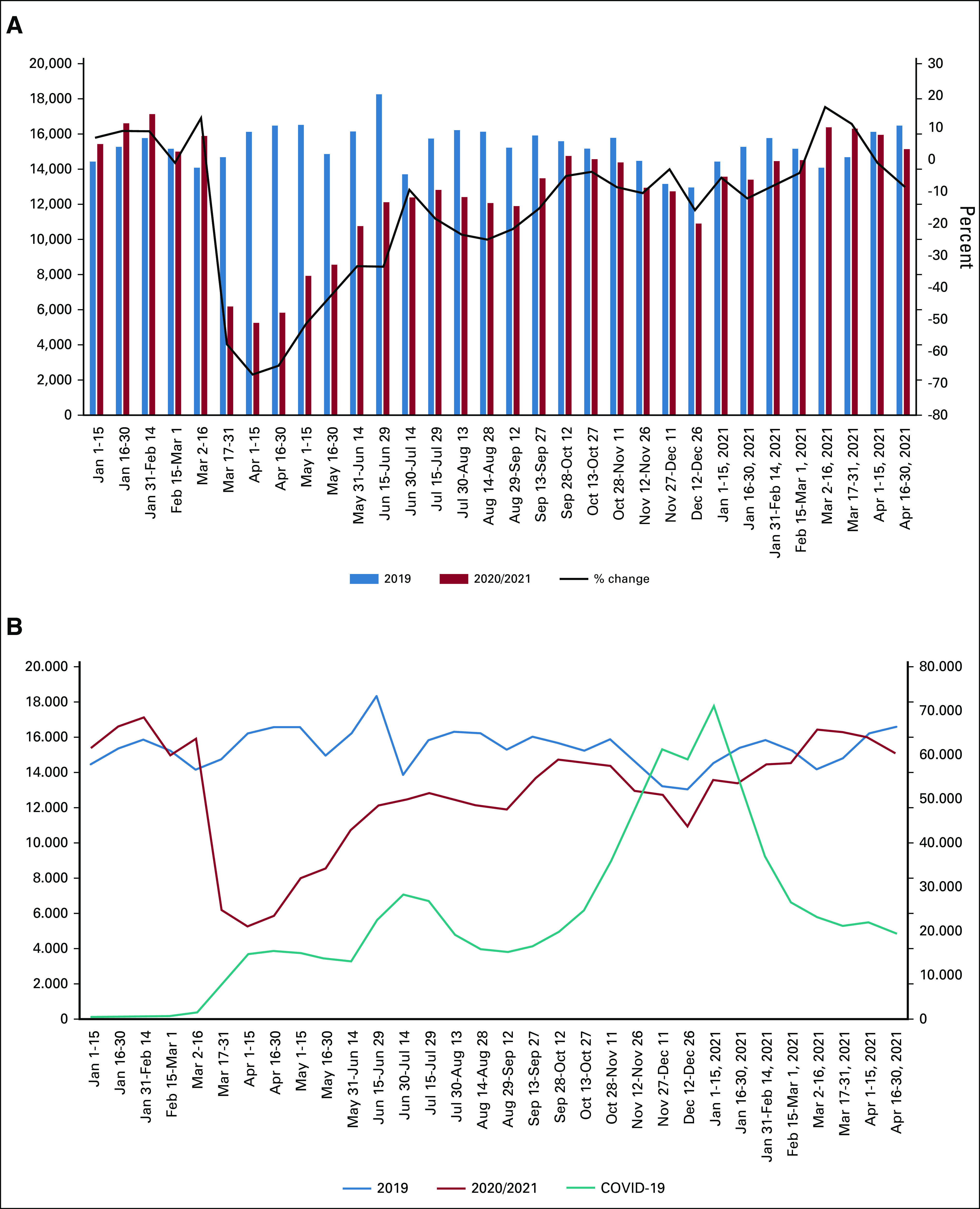
The effects of the COVID-19 pandemic on new incidence patient encounters. (A) New incidence encounters associated with any neoplasm (ie, no previous encounter related to the indicated neoplasm diagnosis). Patients from both 2020 and 2021 compared with 2019 numbers, with the black line representing the percent change as compared with 2019. (B) Plot of patients with new incidence cancer in 2020/2021 compared with 2019 (left ordinate, top two curves) and COVID-19 cases at these institutions superimposed (right ordinate, bottom teal curve).

To quantify the effects of COVID-19 mitigation policies on the cancer patient population, the CCRN was queried for all patients with all neoplasm-related diagnostic codes (C00-D49)—both benign and malignant. Cohorts were generated by 2-week intervals, using January through December 2019 as a basis for prepandemic comparison and the same 2-week intervals in 2020 and 2021 to account for natural seasonal differences in patient presentation. As shown in Figure 2A, after a very precipitous decline of newly diagnosed patients in April 2020 (–67.5% decrease *v* 2019), the presence of patients with new incidence cancer rose steadily, closing the deficit from the previous prepandemic year to –9.6% by the July 4th weekend. Subsequently, the number of patients with new incidence cancer fluctuated from –25% to +16% of 2019 encounters, responding most likely to fluctuations in COVID-19 cases (and particularly the spread of the Delta variant) in various regions of the United States. However, despite this overall upward trend, the number of patients with new incidence cancer remained below the 2019 pre–COVID-19 baseline until March 2021.

In addition, to explore the potential for COVID-19 case levels affecting patients with new cancer, COVID-19 case rates were also examined in the uCCRN (Fig 2B). There appears to be a general correlation between the rise and fall of COVID-19 cases at the network sites and a corresponding opposite fall and rise in new incidence neoplasm patients. This would perhaps reflect the reluctance of people to seek medical diagnostic services during periods of increased COVID-19 activity.

### New Incidence Malignant Cancer Rates

**FIG 3.**
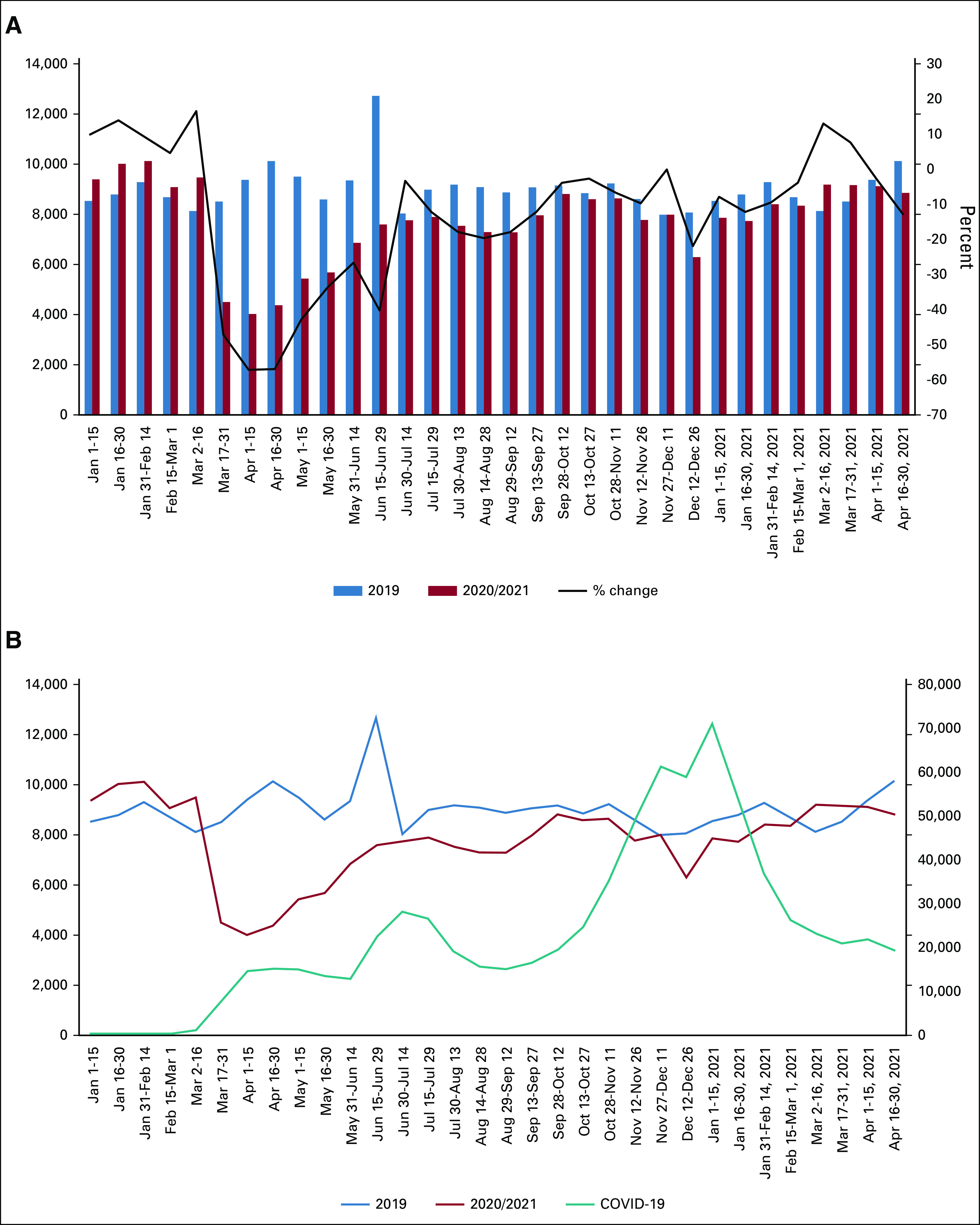
The effects of the COVID-19 pandemic on patients with new incidence encounters associated with malignant neoplasms. (A) With malignant neoplasms (new incidence) defined as no previous encounter related to the indicated diagnosis, patients from both 2020 and 2021 compared with the number of patients in 2019. The black line represents the percent change as compared with 2019. (B) Plot of patients with new incidence malignant cancer in 2020/2021 compared with 2019 (left ordinate, top two curves) and COVID-19 cases at these institutions superimposed (right ordinate, bottom teal curve).

The effect of the continuing pandemic on new incidence cancer patients with only *malignant* disease was also determined (Fig 3A). Similar to the trends seen with all neoplasm (benign and malignant), patients with new incidence malignant disease saw the biggest drop in the initial stages of the pandemic in April 2020 (–57% decrease *v* 2019), and the decreased number of patients continued until March 2021. Importantly, new incidence rates in the 2020 prepandemic were greater than those seen in 2019, suggesting that both 2020 and 2021 would have seen greater numbers of newly diagnosed patients.

Similar to trends seen with new incidence cancer patients with all neoplasms (benign and malignant), the rise and fall of patients with new malignant cancer generally followed the opposite trend in COVID-19 cases, with a large decrease in the beginning of the pandemic in March 2020 (Fig 3B). Taken together, these data indicate that although the number of patients seen for malignant neoplasms for the first time has recovered since the initial drop early in the pandemic, rates have not yet recovered to prepandemic levels. Future studies will be necessary to determine if these trends continue.

### Cancer Screening

**FIG 4.**
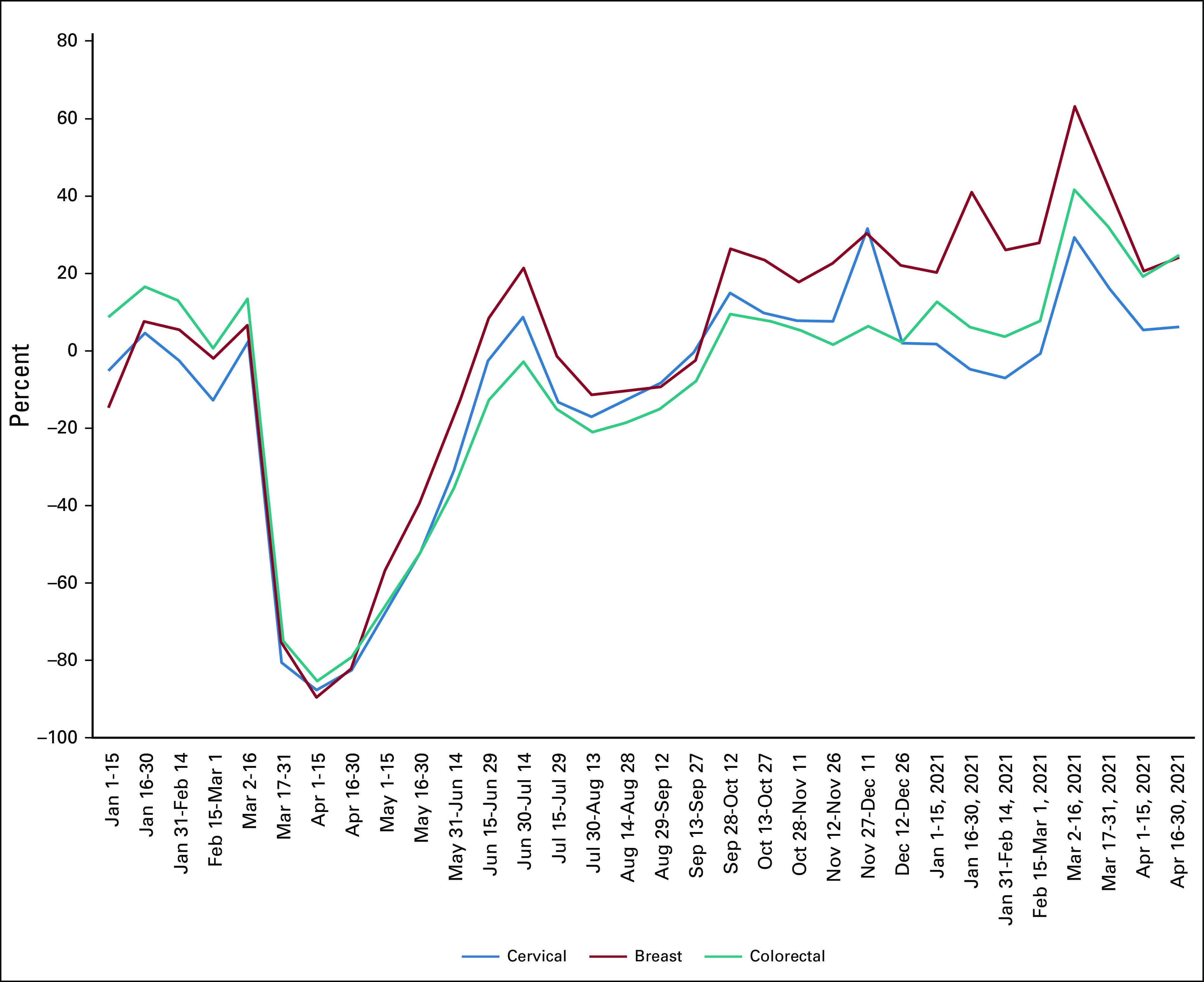
The effects of the COVID-19 pandemic on breast cancer (mammogram), colorectal cancer, and cervical cancer screening. Percent change from the prepandemic year 2019 to 2020-2021 is shown for the indicated 2-week intervals.

In our previous study at the pandemic's onset, we also explored the effects on cancer screening rates for both breast cancer and colorectal cancer. Alarming decreases of –85% to –90% were observed for both screening examinations (Fig 4). These results were similar to a study from the EPIC Health Research Network, using institutions on the EPIC EMR System, where breast and colorectal cancer screenings were shown to decline by more than 90% in March and April.^5^ Concern was expressed for a potential increase in the presentation of later-stage disease for newly diagnosed patients with breast and colon cancers in future months. However, as with the numbers of patients with newly incidence cancer, the numbers of breast and colorectal screenings approached or exceeded 2019 numbers by the July 4th weekend (breast screening, +21%; colorectal, –3%). There were similar fluctuations in screening numbers as with new incidence patients for the remainder of 2020 into 2021, but the numbers exceeded the prepandemic base year from fall 2020 onward. For this study, we also obtained numbers for cervical screening. As seen in Figure 4, cervical screening trends generally followed those of breast and colorectal cancer screenings. Interestingly, although breast cancer screening rates increased to levels above prepandemic rates by September 2020 (and remained higher), colorectal and cervical cancer rates remained similar to the 2019 levels, suggesting that the cancer screenings missed because of the early stages of the pandemic have not been recovered.

### Initial Cancer Stage

To evaluate whether any upstaging occurred for patients with breast and colon cancer after the drastic decrease in screenings for these diseases in March and April 2020, we queried for the initial cancer stage of these patients in subsequent months. These data are typically available from institutional cancer registries (tumor registries), not EMRs. Given the typical lag in reporting data to the cancer registries, data were only present for December 2020 and only for about one third of the network institutions. *Conclusions on the basis on these data must therefore be considered preliminary*.

The distributions in 2019 and 2020 of initial diagnostic stages of both breast and colon cancers (Table 1) were compared using the chi-square statistical test of independence. The statistical analysis showed that *there is NO significant difference between 2019 and 2020 in the stage distributions of patients with new incidence breast cancer* (χ^2^ = 3.44, *df* = 3, *P* > .10) *or colon cancer* (χ^2^ = 2.33, *df* = 3, *P* > .10). Since all chi-square values are < 1.0, all observed counts are as expected. The percent distribution columns for 2019 and 2020 show no glaring variances (all differences < 2%).

**TABLE 1.**
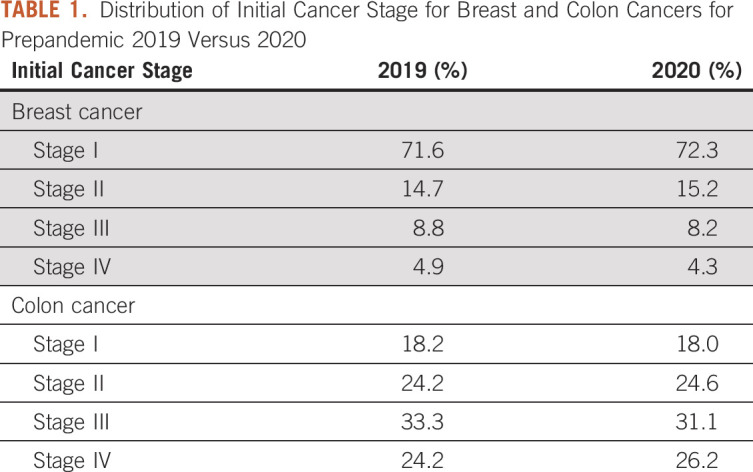
Distribution of Initial Cancer Stage for Breast and Colon Cancers for Prepandemic 2019 Versus 2020

## DISCUSSION

In an April 2020 commentary, Mazer^6^ suggested that the suspension of cancer screening because of the pandemic would provide a real-world experiment on the efficacy of screening. However, the subsequent immediate resumption of screening was felt to prematurely shut down the experiment before any conclusions could be derived.

There have been a few reports to date on the effect of the suspension of cancer screening on initial cancer diagnoses. A national physician survey of radiation oncologist practice leaders by the American Society for Radiation Oncology (ASTRO)^7^ had two thirds of the respondents reporting new patients presenting with more advanced disease compared with that before the pandemic. The survey response rate was 23%. At the University of Cincinnati, COVID-19 caused a significant disruption in low-dose computed tomography lung cancer screening. The percentage of patients with lung nodules suspicious for malignancy were significantly increased after screenings resumed (8% *v* 29%; *P* < .01).^8^ However, given the limited follow-up period, not all suspicious lung nodules were confirmed as lung malignancy. A study^9^ reporting data from a hospital in Modena, Italy, showed a significant decrease (–10%) in *in situ* breast cancer diagnosis, but an increase of approximately 11% in node-positive and stage III cancer after a 2-month stop in breast cancer screening followed by a resumption to a reduced level of screening. It should be noted that the reported increase was limited to the 3 months (May-July 2020) subsequent to the March-April screening suspension.

Although the Cincinnati and Modena studies involved single institutions, a study^10^ in the Netherlands reported on the effect of the closure of the Dutch national breast screening program in March-April 2020. New incidence breast cancer diagnoses fell by 37% during the interval March-August 2020 compared with prior years of 2018-2019. As of August 2020, at which time the breast screening program was at 60% capacity, the incidence of lower-stage tumors mainly decreased and no shift toward a higher tumor stage at diagnosis was seen.

Our study was similar to the Dutch study in that it was multi-institutional and did not observe increased initial diagnostic stage subsequent to the drastic dip in breast and colorectal screening. Furthermore, our study included data for 8 months after the March-April hiatus by which time screening activities had resumed to prior levels. No appreciable change in initial cancer stage is observed for these diseases for which the roughly 2-month hiatus in cancer screening was followed by a return to normal rates and a compensatory increase in screening numbers as postponed screenings were rescheduled. That an increase in initial stage did not occur for these diseases is probably because the interruption in screening was brief and quickly ended.

In conclusion, despite the fluctuations in the number of COVID-19 cases as the pandemic ensued in the United States in 2020 and the first 4 months of 2021, the steep declines observed during March and April 2020 in screening for patients with breast and colon cancer and patients with newly diagnosed cancer did not continue. Screening and new incidence cancer numbers quickly rose to prepandemic levels. The concern that more patients with advanced-stage cancer would be seen in the months following the drastic dips of March-April 2020 was not realized as the major disruption to normal cancer care was limited to these 2 months. Although there has been much frustration over the failure to end the COVID-19 pandemic even in the presence of effective vaccines, this study shows that the diagnosis of patients with cancer has continued in a reasonable manner.
